# Supplementary material for: Modeling glioblastoma heterogeneity as a dynamic network of cell states
Source: Mol Syst Biol. 2021 Sep 16;17(9):e10105. doi: 10.15252/msb.202010105 (PMC8444284; doi:10.15252/msb.202010105)
Supplement: Supplementary file 6 — Source Data for Figure 5 [file MSB-17-e10105-s004.zip › Figure5A_sourcedata/GSEA_3071/hallmarks_stateII.GseaPreranked.1623742536692/HALLMARK_FATTY_ACID_METABOLISM.html]

Details for gene set HALLMARK\_FATTY\_ACID\_METABOLISM[GSEA]

|  || Dataset | state23071 |
| Phenotype | NoPhenotypeAvailable |
| Upregulated in class | na\_neg |
| GeneSet | HALLMARK\_FATTY\_ACID\_METABOLISM |
| Enrichment Score (ES) | -0.15734075 |
| Normalized Enrichment Score (NES) | -0.7189074 |
| Nominal p-value | 0.83154124 |
| FDR q-value | 0.94151866 |
| FWER p-Value | 1.0 |
Table: GSEA Results Summary

  

Fig 1: Enrichment plot: HALLMARK\_FATTY\_ACID\_METABOLISM      
 Profile of the Running ES Score & Positions of GeneSet Members on the Rank Ordered List

  

| PROBE | GENE SYMBOL | GENE\_TITLE | RANK IN GENE LIST | RANK METRIC SCORE | RUNNING ES | CORE ENRICHMENT || 1 | NCAPH2 |  |  | 207 | 0.868 | -0.0117 | No |
| 2 | MGLL |  |  | 227 | 0.848 | 0.0221 | No |
| 3 | YWHAH |  |  | 273 | 0.781 | 0.0464 | No |
| 4 | ODC1 |  |  | 430 | 0.664 | 0.0380 | No |
| 5 | ACAT2 |  |  | 446 | 0.653 | 0.0640 | No |
| 6 | SMS |  |  | 608 | 0.575 | 0.0503 | No |
| 7 | HADH |  |  | 706 | 0.530 | 0.0505 | No |
| 8 | ME1 |  |  | 835 | 0.489 | 0.0411 | No |
| 9 | FH |  |  | 849 | 0.485 | 0.0599 | No |
| 10 | ADSL |  |  | 991 | 0.446 | 0.0453 | No |
| 11 | HSP90AA1 |  |  | 1008 | 0.443 | 0.0615 | No |
| 12 | GPD2 |  |  | 1202 | 0.397 | 0.0319 | No |
| 13 | DHCR24 |  |  | 1253 | 0.386 | 0.0371 | No |
| 14 | HSDL2 |  |  | 1326 | 0.373 | 0.0362 | No |
| 15 | LDHA |  |  | 1374 | 0.361 | 0.0410 | No |
| 16 | ECI2 |  |  | 1516 | 0.338 | 0.0216 | No |
| 17 | CBR3 |  |  | 1522 | 0.336 | 0.0356 | No |
| 18 | HSPH1 |  |  | 1595 | 0.324 | 0.0326 | No |
| 19 | KMT5A |  |  | 1787 | 0.300 | -0.0009 | No |
| 20 | UGDH |  |  | 1797 | 0.299 | 0.0104 | No |
| 21 | S100A10 |  |  | 1852 | 0.292 | 0.0104 | No |
| 22 | RAP1GDS1 |  |  | 1890 | 0.287 | 0.0142 | No |
| 23 | CPT1A |  |  | 1959 | 0.278 | 0.0101 | No |
| 24 | ACSL4 |  |  | 2006 | 0.272 | 0.0111 | No |
| 25 | BLVRA |  |  | 2152 | 0.257 | -0.0131 | No |
| 26 | PCBD1 |  |  | 2255 | -0.254 | -0.0267 | No |
| 27 | BPHL |  |  | 2371 | -0.267 | -0.0429 | No |
| 28 | PSME1 |  |  | 2413 | -0.272 | -0.0407 | No |
| 29 | HADHB |  |  | 2489 | -0.283 | -0.0463 | No |
| 30 | ECH1 |  |  | 2885 | -0.353 | -0.1277 | No |
| 31 | GSTZ1 |  |  | 2948 | -0.364 | -0.1265 | No |
| 32 | PPARA |  |  | 3067 | -0.389 | -0.1379 | Yes |
| 33 | UROS |  |  | 3092 | -0.396 | -0.1259 | Yes |
| 34 | ACOX1 |  |  | 3121 | -0.404 | -0.1145 | Yes |
| 35 | ACAA1 |  |  | 3203 | -0.429 | -0.1150 | Yes |
| 36 | DECR1 |  |  | 3261 | -0.444 | -0.1089 | Yes |
| 37 | SERINC1 |  |  | 3324 | -0.465 | -0.1031 | Yes |
| 38 | G0S2 |  |  | 3545 | -0.568 | -0.1316 | Yes |
| 39 | ALDH3A2 |  |  | 3609 | -0.609 | -0.1195 | Yes |
| 40 | GABARAPL1 |  |  | 3667 | -0.657 | -0.1038 | Yes |
| 41 | IDH1 |  |  | 3842 | -0.832 | -0.1089 | Yes |
| 42 | PTPRG |  |  | 3958 | -1.083 | -0.0882 | Yes |
| 43 | GPD1 |  |  | 4092 | -2.700 | 0.0015 | Yes |
Table: GSEA details [plain text format]

  

Fig 2: HALLMARK\_FATTY\_ACID\_METABOLISM: Random ES distribution      
 Gene set null distribution of ES for **HALLMARK\_FATTY\_ACID\_METABOLISM**
